# Factors and Causes of Puerperal Sepsis in Kilimanjaro, Tanzania: A Descriptive Study among Postnatal Women who Attended Kilimanjaro Christian Medical Centre

**DOI:** 10.24248/eahrj.v4i2.639

**Published:** 2020-11-26

**Authors:** Debora C. Kajeguka, Neema Reuben Mrema, Akili Mawazo, Rosemary Malya, Maseke R. Mgabo

**Affiliations:** a Faculty of Medicine, Department of Microbiology and Immunology, Kilimanjaro Christian Medical University College, Moshi, Tanzania; b Faculty of Medicine, Department of Microbiology and Immunology, School of Medicine, Muhimbili University of Health and Allied Sciences, Dar es Salaam, Tanzania; c Department of Nursing, Kilimanjaro Christian Medical University College, Moshi, Tanzania; d Department of Population Studies, Institute of Rural Development Planning

## Abstract

**Background:**

Puerperal sepsis is the major cause of maternal morbidity and mortality worldwide. About 94% of maternal mortality occur in low and middle-income countries including Tanzania.

**Objective:**

To estimate the prevalence, document factors and causes of puerperal sepsis among postnatal women who attended postnatal care in Kilimanjaro Christian Medical Centre Hospital in the year 2015.

**Methodology:**

A descriptive cross-sectional study was carried out at Kilimanjaro Christian Medical Centre, Tanzania. A total of 183 medical records of attendance in 2015 were used for the study. Information about the isolated organism in culture was retrieved from the Laboratory Information System.

**Results:**

The prevalence of puerperal sepsis was 11.5% (21/183). The most common factors and causes of puerperal sepsis included caesarean section 66.7% (14/21), postpartum haemorrhage 57.1% (12/21), moderate to severe anaemia 61.9% (13/21), prolonged labour 76.2% (16/21) and bacterial infection 90.5% (19/21). The difference was significant at p<.05. The most bacteria species isolated among women with puerperal sepsis was Staphylococcus spp 50.0% (7/14), Escherichia 28.6% (4/14) and Streptococcus spp 21.4% (3/14).

**Conclusion:**

Puerperal sepsis is prevalent (11.5%) at Kilimanjaro Christian Medical Centre. *Staphylococcus spp* was found to be a predominant isolate which causes puerperal sepsis followed by *E. coli* and *Streptococcus spp*.

## BACKGROUND

Puerperal sepsis is the major cause of maternal morbidity and mortality worldwide while about 94% of maternal mortality occur in low and middle-income countries.^1^ Puerperal sepsis is among the preventable conditions in all settings.^2^ World health organization (WHO) defines puerperal sepsis as infection of the genital tract occurring at any time between the onset of rupture of membranes or labour and the 42 days (6 weeks) after delivery in which 2 or more of the following are present: pelvic pain, fever, abnormal vaginal discharge, abnormal smell/foul odour discharge or delay in uterine involution.^3^ Puerperal sepsis causes 10.7% of maternal deaths and it is one of the 5 common causes of maternal mortality worldwide.^4^ Despite major advances in postnatal care, puerperal sepsis remains a common and potentially preventable cause of direct maternal death.^5–8^ Risk factors for puerperal sepsis include retained products of conception, chorioamnionitis, pelvic abscess, and wound infection are the common causes for severe puerperal sepsis and septic shock in pregnancy and puerperium.^5^

It is reported that major consequences of puerperal sepsis are chronic or acute pelvic inflammatory disease, bilateral tubal occlusion and infertility.^9,10^ After delivery there is susceptibility to invasion of the birth canal by microorganisms for several days which may lead to occurrence of puerperal sepsis when there are births in unhygienic conditions, prolonged rupture of membranes, prolonged labour, postpartum haemorrhage and when vagina examination is done frequently during labour i.e when examinations are done more than 5 times.^11^

Puerperal sepsis is evidenced with clinical signs such as fever above 38°C, pelvic pain, delayed reduction of the uterine size and smelling vaginal discharge.^9,12^ Most puerperal sepsis is due to infection of the genital tract by pathogens that colonise the cervix and vagina, gain access to amniotic fluid and invade the devitalised uterine tissues.^13^. Bacteria that cause puerperal sepsis includes, *Streptococci* spp, *Staphylococcus* spp, *Escherichia coli, Clostridium tetani, Clostridium welchii, Chlamydia* spp and *Gonococcus* spp.^13^ Moreover, several studies reported the common aetiologies for puerperal sepsis, including *Klebsiella* spp, *E .coli, S. aureus, Pseudomonas* and *Enterococci*.^14–18^

In Tanzania, few studies have been conducted on puerperal sepsis. The findings have registered a varied puerperal sepsis prevalence ranging from 20% to 30%.^19,20^ These two studies have been done in Dar Es Salaam. A previous study done in Mwanza reported a prevalence of 38.9%.^21^. In the year 2015, the average national puerperal sepsis prevalence in Tanzania was 29.7%^22^ which is twice as much as the global puerperal sepsis prevalence which is estimated to be 11%.^23^. To this end, it is therefore important to estimate the prevalence, document factors and causes of puerperal sepsis among postnatal women at Kilimanjaro Christian Medical Centre (KCMC) hospital. The information will help to establish baseline information and identify the gap that can be used during intervention and control of puerperal sepsis. Scarcity of information regarding puerperal sepsis has led to negligence of puerperal sepsis whilst increasing incidences of maternal mortality. The research questions is “what are the factors and causes of puerperal sepsis among postnatal women?”.

## METHODOLOGY

### Study Design and Area

This was a descriptive cross-sectional study conducted at KCMC hospital. KCMC is referral hospital located in Kilimanjaro region, Tanzania. Kilimanjaro region is found at the foothills of Mount Kilimanjaro, (www.kcmc.ac.tz). The region has a population of 1,640,087, with average annual population growth rate of 1.8 %, Fertility Rate (TFR) for Kilimanjaro Region is 4.3 (Adjusted) persons per woman in 2013 and Child-woman ratio of 0.46, maternal mortality rate in Kilimanjaro is 492.1/100,000 live births.^24,25^

The Department of Obstetrics at KCMC serves as a zonal referral centre for complicated obstetric patients. The hospital has an average annual delivery of 3,300, of which 33% are caesarean deliveries. The labour ward has 4 beds partitioned along the sterile room, with the sterile corridor serving for the extras. There are two operating theatres along the labour ward used for caesarean section services and other obstetric surgical emergencies.^25^

### Sample Size Estimation

The sample size for this study was calculated using the following formula n=z^2^pq/d^2^. In this equation, *n* is the sample size, *z* is the value of the Standard Normal Distribution at 5% level (1.96), *p* is the prevalence, *q* = 1–*p*, and *d* is the precision level (0.05). The prevalence of puerperal sepsis is 12%.^26^. Sample size=1.96^2^*0.12*(1-0.12)/(0.05)^2^. The sample size was 178+10%=183.

### Sampling Techniques and Inclusion/Exclusion Criteria

A systematic sampling technique was used to select the files. The hospital has annual delivery of 3,300, a list of all deliveries in 2015 was requested from the KCMC medical records department. The sampling interval was after every 18^th^ delivery. The study included patient files from January 2015 to December 2015, patients who visited KCMC hospital for obstetrics and gynaecology services. All files with incomplete information were excluded from the study.

### Data Collection Method

Data was collected from the Medical Record Department. Information about isolated causative organism was retrieved from the Laboratory Information System. All required data relevant for the study was recorded using a constructed data extraction sheet. Demographic information such as age, marital status and residence were recorded. Other information such as Haemoglobin level, postpartum haemorrhage, prolonged labour, diabetes, HIV status, mode of delivery and type of bacteria isolated were collected.

### Data Analysis

Data was collected and entered in an excel sheet. Quantitative data was analysed using Statistical Package for Social Science (SPSS) version 22 software (SPSS Inc., Chicago, IL, USA). Descriptive statistical analysis was done to test the effect of each factor on the outcome. Chi-square (χ2) was used to compare categorical data while Fisher's exact test was used in cases when expected counts were less than 5. A *P*-value < .05 was considered statistically significant.

#### Definitions

Mild anaemia was defined as haemoglobin concentration <11.0-11.9 g/dl. Moderate to severe anaemia was defined as haemoglobin concentration ≤ 10.9 g/dL.^27^

### Ethical Consideration

Ethical approval was obtained from the Kilimanjaro Christian Medical University College Research and Ethics Review Committee (CRERC) with certificate number 2072. Privacy and confidentiality were ensured since the information from patients’ file records is kept confidential. Patient names and their corresponding file numbers were not used but instead, individual files identification numbers were generated and used in the data extraction sheet.

## RESULTS

### Demographic Characteristics and Prevalence of Puerperal Sepsis

A total of 183 files were reviewed. Most of the participants 73 (39.9%) were aged between 26 and 35 years, with a mean (±SD) age of 30.63 (±8.08). The majority of participants were married 143 (78.1%) and 93 (50.8%) were residing in rural areas. Furthermore, 86(47%) were multigravid. The prevalence of puerperal sepsis was 11.5% (21/183) Table 1.

**TABLE 1:** Demographic Characteristics of the Participants (N=183)

Variable	Frequency (n)	Percent (%)
**Age category**		
17 – 25 years	55	30.1
26 – 35 years	73	39.9
36 – 48 years	55	30.1
**Marital status**		
Single	36	19.7
Married	143	78.1
Widow	4	2.2
**Residence**		
Rural	93	50.8
Urban	90	49.2
**Parity**		
Primigravid	66	36.1
Multigravid	86	47.0
Grand multigravid	31	16.9

### Bacteria Isolated as Causative Agent of Puerperal Sepsis

Among 21 patient files with puerperal sepsis, 14 had results of the isolated causative organism, 2 files had only report of gram stain (gram-negative rods) and 4 files had no record of any isolated organism. The most bacteria species (*spp*) isolated among women with puerperal sepsis was *Staphylococcus spp* 50% (7/21), *Escherichia* 28.6%

**FIGURE 1.**
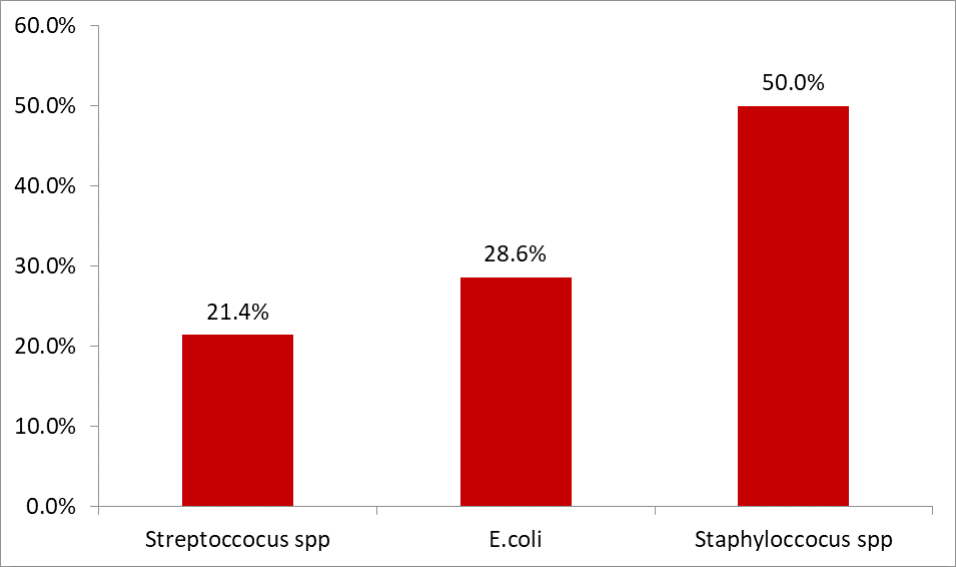
Common Isolated Bacteria as Causative Agent of Puerperal Sepsis

### Factors and Causes of Puerperal Sepsis

In this study, the most common factors associated with puerperal sepsis included mode of delivery (caesarean section), postpartum haemorrhage, moderate to severe anaemia, prolonged labour and bacterial infection. There was significant difference in puerperal sepsis proportions among women who had Spontaneous Vaginal Delivery (SVD) 33.3% (7/21) as compared to those with caesarean section 66.7% (14/21), *χ*^2^ =18.49, *p = ≤.01*. Women who did not experiences Postpartum Haemorrhage 57.1% (12/21) were more frequent as compared to those who did not have 42.9% (9/21), *χ*^2^ =2 5.85, *p<.001*. The study found that more women with moderate to severe anaemia 61.9% (13/21) were having puerperal sepsis as compared to others, Fischer exact=8.40, *p =.01*. The study shows that puerperal sepsis were more prevalent among women who experienced prolonged labour 76.2% (16/21) as compared to those who did not experience 23.8% (5/21), *χ*^2^=64.77, *p<.001*. Women with puerperal sepsis were more infected with bacteria 90.5% (19/21) as compared to other cause of infection 9.5% (2/21) such as *Candida spp, χ*^2^=162, *p<.001*. Other factor such as age, residence, parity, pelvic infection during pregnancy, HIV status and Diabetes were not associated with puerperal sepsis, Table 2.

**TABLE 2:** Malaria Prevalence by Age Category

Variable	With Puerperal Sepsis (N=21) %(n)	Without Puerperal Sepsis (N=162) %(n)	Chi-square	P-value
**Age category**				
17 – 25 years	23.8 (5)	30.9 (50)	0.8	.6
26 – 35 years	38.1 (8)	40.1 (65)		
36 – 48 years	38.1 (8)	29.0 (47)		
**Residence**				
Urban	57.1 (12)	48.1 (78)	0.6	.4
Rural	42.9 (9)	51.9 (84)		
**Parity**				
Primigravid	28.6 (6)	37.0 (60)	1.03	.5
Multigravid	47.6 (10)	46.9 (76)		
Grand multigravida	23.8 (5)	16.0 (26)		
**Mode of delivery**				
SVD	33.3 (7)	77.8 (126)	18.49	≤.001
Caesarean section	66.7 (14)	22.2 (36)		
**Postpartum Haemorrhage**				
Yes	57.1 (12)	12.3 (20)	30.89	≤.001
No	42.9 (9)	87.7 (142)		
**Anaemia**				
Normal	33.3 (7)	57.4 (93)		.01^*^
Mild anaemia	4.8 (1)	12.3 (20)		
Moderate to severe anaemia	61.9 (13)	30.2 (49)		
**Pelvic infections during pregnancy**				
Yes	38.1 (8)	32.1 (52)	0.3	.5
No	61.9 (13)	67.9 (110)		
**HIV Status**				
Positive	9.5 (2)	3.6 (6))		.2^*^
Negative	90.5 (19)	96.3 (155)		
**Prolonged labour**				
Yes	76.2 (16)	8.0 (13)	64.77	<.001
No	23.8 (5)	92.0 (149)		
**Diabetes**				
Yes	4.8 (1)	1.2 (2)	-	.3^*^
No	95.2 (20)	98.8 (160)		
**Bacterial Infection**				
Yes	90.5 (19)	0.0 (0)	162	<.001
No	9.5 (2)	100 (161)		

* Fischer exact test, SVD: Spontaneous Vaginal Delivery

## DISCUSSION

The study aimed to estimate the prevalence, document factors and causes of puerperal sepsis among postnatal women who attended postnatal care in Kilimanjaro Christian Medical Centre Hospital in the year 2015. The prevalence of puerperal sepsis was 11.5%. The findings are relatively comparable with two studies conducted at Muhimbili, Tanzania where the prevalence was 9.2% in 2011^28^ and 11.2% in 2019.^14^ As well as compared to a recent countrywide study which reported a higher prevalence of 16.7%.^29^

In the present study, 50% of commonly isolated bacteria were *Staphylococcus spp*. The results are higher than other reported findings at Muhimbili, Tanzania (22.7%)^14^ and Sudan (39.5%).^16^ The high prevalence of *Staphylococcus spp* as the cause of puerperal sepsis might be exogenous where pathogens from nearby skin flora or contact with contaminated non-sterilised instruments or frequent vaginal examination with unwashed hands.^30^ Additionally, the current study did not go further to characterise the Staphylococcus at specie level, although *Staphylococcus aureus* is more expected than other species. However, in other settings, *Staphylococcus epidermidis* were isolated among women with puerperal sepsis in other settings.^30^ Although, *S. epidermidis* is rarely reported but it is a significant nosocomial pathogen, patients may acquire infection when they have compromised immunity.^30^

The present study reports that *E.coli* (28.6%) as the second common cause of puerperal sepsis. This is similar to a study conducted in Harare, which reported *E.coli* as the commonest cause of puerperal sepsis.^15^ A study conducted at Muhimbili also reported *E. coli* as one of the causes of puerperal sepsis, accounting for 27.3% of all isolates.^14^ Variations in the proportion of the commonest bacteria as the cause of puerperal sepsis may be due to differences in the immune status of an individual or population of commensal bacteria.^31^ Furthermore, the differences could be due to study design used, settings as well as variations in bacteriological culture techniques used.^2,32^ It is recommended that in order to have proper infection control measures, it is required that there must be proper education, improvements of guidelines and various technologies and introduction of new clinical guidelines^33^, and continuous improvement of all aspects of maternal health.^34^

In this study, various factor were associated with puerperal sepsis including mode of Delivery (caesarean section), Postpartum Haemorrhage, moderate to severe anaemia and prolonged Labour were statistically associated with puerperal sepsis. Mode of delivery was significantly associated with puerperal sepsis. Data shows that mothers who delivered by SVD were associated with puerperal sepsis when compared to those delivered by caesarean section. The findings are different from a study conducted in Ethiopia^2^ and Nigeria.^18^ Moreover, this is different from a study conducted in Uganda, which reported that Caesarean delivery was independently associated with puerperal sepsis.^32^ This is inconsistent with the study conducted in Ethiopia which reported that participants associated with caesarean section were more less likely to develop puerperal sepsis when compared to those who gave birth through SDV.^4^ In this study, prolonged labour was associated to puerperal sepsis. This is in line with a study by Demisse *et al.,* which reported that participants who experienced labour for 12 to 24 hours and more than 25 hours were 3.1 and 4.7 times respectively more likely to develop puerperal sepsis as compared to those who experienced labour for less than 12 hours.^2^

The present study also revealed that moderate to severe anaemia is among factors that are associated with puerperal sepsis. In Kenya, a study was conducted and reported that anaemia is indirectly associated with puerperal sepsis as well as maternal mortality.^35^ Further research is required to explore the role of anaemia to puerperal sepsis. The study provides valuable information that is needed for planning meaningful Reproductive Health Control Programs that aim at reducing the prevalence and associated morbidity of puerperal sepsis among postnatal women attending care at the Hospital.

There is a need for educating the community on hygiene practices especially for postnatal women to reduce cases of puerperal sepsis. Health care providers should be emphasised on working under aseptic conditions to prevent nosocomial infections. During the attendance of antenatal care clinics, hygiene and proper nutrition during and after pregnancy should be taught and emphasised, women should also be enlightened about puerperal sepsis risks and preventive measures.

### Strength and Limitation of the Study

These data signify unique and vigorous information from Kilimanjaro and contribute to the available knowledge on puerperal sepsis among postnatal women in Kilimanjaro region. However, the study is limited with the following; first, the results are limited to Hospital settings, the prevalence and risks of the disease in the community might be different. Secondly, the study was conducted on a small sample size. This may have provided bias on the prevalence and risk of puerperal sepsis, a study with a larger sample size is required.

## CONCLUSION

Puerperal sepsis is prevalent at KCMC hospital. The common risk factors for puerperal infection includes mode of delivery, Postpartum Haemorrhage, prolonged labour, and anaemia. *Staphylococcus spp* was found to be a predominant isolate which causes puerperal sepsis followed by *E. coli* and *Streptococcus spp*.
